# Dual Stimuli-Responsive Orthodontic Aligners: An In Vitro Study

**DOI:** 10.3390/ma16083094

**Published:** 2023-04-14

**Authors:** Dennis Schönfeld, Samantha Koss, Nils Vohl, Fabian Friess, Dieter Drescher, Thorsten Pretsch

**Affiliations:** 1 Fraunhofer Institute for Applied Polymer Research IAP, Geiselbergstr. 69, 14476 Potsdam, Germany; 2 Department of Orthodontics, Universitätsklinikum Düsseldorf, Moorenstr. 5, 40225 Düsseldorf, Germany

**Keywords:** aligner therapy, shape memory polymer, thermo-responsive, water-responsive, thermoplastic polyurethane

## Abstract

Aligner therapy for orthodontic tooth movement is gaining importance in orthodontics. The aim of this contribution is to introduce a thermo- and water-responsive shape memory polymer (SMP), which could lay the foundation for a new type of aligner therapy. The thermal, thermo-mechanical, and shape memory properties of thermoplastic polyurethane were studied by means of differential scanning calorimetry (DSC), dynamic mechanical analysis (DMA), and various practical experiments. The glass transition temperature of the SMP relevant for later switching was determined to be 50 °C in the DSC, while the tan δ peak was detected at 60 °C in the DMA. A biological evaluation was carried out using mouse fibroblast cells, which showed that the SMP is not cytotoxic in vitro. On a digitally designed and additively manufactured dental model, four aligners were fabricated from an injection-molded foil using a thermoforming process. The aligners were then heated and placed on a second denture model which had a malocclusion. After cooling, the aligners were in a programmed shape. The movement of a loose, artificial tooth and thus the correction of the malocclusion could be realized by thermal triggering the shape memory effect, at which the aligner corrected a displacement with an arc length of approximately 3.5 mm. The developed maximum force was separately determined to be about 1 N. Moreover, shape recovery of another aligner was realized within 20 h in 37 °C water. In perspective, the present approach can help to reduce the number of orthodontic aligners in therapy and thus avoid excessive material waste.

## 1. Introduction

Aligners that allow sequential tooth movement are commonly used in orthodontic treatment today. The so-called staging of orthodontic tooth movements essentially means that the intended movement of the teeth is performed sequentially with aligners [1]. In principle, the use of aligners requires good oral hygiene. It is not uncommon for children to still be dependent on their parents for support [2]. From a functional point of view, aligners are well-suited to correct mild to moderate malocclusions. However, the correction capacity of a single aligner is very limited. Only movements of up to 0.2 mm and rotations of up to 3° per aligner are feasible without plastic deformation of the employed polymer material [3]. Studies have recommended no greater linear movements than 0.2 mm or rotations of less than 1.5° in order to avoid overloading of the periodontal tissue [4,5]. Consequently, about 30 aligners each for the upper and lower dental arch [6] may be necessary to be produced to bring a classic orthodontic treatment to a successful outcome.

Aligners are produced from polymer foil via vacuum forming and the same number of dental arches is printed for the thermoplastic forming process. Consequently, large amounts of plastic waste are produced. To date, the availability of materials for aligner therapy is limited. Most often, “static” materials with clearly defined physical properties such as polyethylene terephthalate (PET), polyethylene terephthalate glycol (PET-G), and specific types of polyurethane (PU) [7,8] are used. Some manufacturers such as Straumann^®^ introduced multi-layer material approaches, such as foil made from PET-G with PU embedded in them [9] to optimize the mechanical behavior and avoid high initial and non-physiological forces acting on the teeth. However, multi-layer materials are costly to manufacture and are less sustainable against the prospect of recycling individual materials, while therapy requires an ordinary number of aligners.

To overcome these drawbacks, approaches using highly functional materials, such as shape memory polymers (SMPs), offer interesting possibilities [10,11,12,13,14]. SMPs can make contributions in various fields of work such as in biomedicine [15,16,17,18,19,20,21], sensor applications [22,23,24], and soft robotics [25,26,27]. They are able to retain an imposed temporary shape after a thermomechanical treatment, so-called “programming”, until the shape memory effect (SME) is triggered, whereupon the material returns to its thermally processed permanent shape. Shape recovery of the polymer is due to the gain in entropy, according to the principle of entropy elasticity [11]. The SME is commonly triggered by heat [15,22,28,29,30,31], but if suitable fillers are used, other methods such as indirect heating by magnetic [32,33,34] or electric fields [35,36,37,38] can also be applied. Beyond that, changes in humidity [39,40,41,42] and pH value [43,44] may also trigger the SME. Some SMPs may combine two stimuli-responsivities in one material system. One example is a urethane-based SMP built up by poly(ethylene glycol) (PEG), poly(ε-caprolactone), and poly(dimethylsiloxane) [45]. Apart from the thermo-responsive SME, the water-responsive SME was realized by the solvation and recrystallization of the PEG switching segment. To address the same combination of material functionalities, Wang et al. developed a shape memory polyurethane, in which telechelic-hydroxylated polyhydroxyalkanoate and PEG were used as switching segments [46]. However, solutions relying on semi-crystalline polyurethane-based SMPs are often unsuitable for applications such as aligners where optical transparency is required.

If SMPs are used as functional materials for orthodontic aligners, this may open up promising possibilities for therapeutic treatment. For instance, the SME of a programmed aligner could be triggered intraorally to realize tooth motion. On the other hand, minor shape adjustments by controlled heating, for instance, can be carried out outside the oral cavity in a targeted manner before an aligner is used. In any case, the ability of an SMP to recover shape on demand could reduce the number of required staging steps in an orthodontic treatment compared to therapies with common aligner materials. Some scientific works describe the use of SMPs as orthodontic archwires in multibracket systems [47,48,49,50]. However, only a few contributions can be found in the literature regarding technology developments for aligner treatment. Recently, Elshazly et al. conducted tooth movement on a typodont model by using a clear aligner made of SMP in an in vitro study [51]. They showed that repositioning movements of the upper left central incisor tooth were possible. In another contribution, Elshazly et al. investigated the forces of a splint while triggering the SME [52]. In both cases, no material analysis was conducted to gain detailed insights into the behavior of the employed commercially available SMP.

In this study, an SMP belonging to the class of thermoplastic polyurethanes (TPUs) is reported. It will be demonstrated that the poly(ether urethane) is both thermo- and water-responsive, thus exhibiting dual stimuli-responsiveness, and the results of a biological evaluation with respect to in vitro cytotoxicity will be provided. Finally, it will be shown for programmed aligners how in vitro the correction of a tooth malposition in an artificial dental arch is possible in one step by triggering the SME either thermally or induced by the uptake of water at 37 °C.

## 2. Materials and Methods

*Materials:* Desmophen 1262 BD was kindly provided by Covestro Deutschland AG (Leverkusen, Germany). It is a linear polypropylene polyether glycol (PPG) with a molecular weight of about 430 g mol^−1^ [53]. Titanium(IV)bis(acetylacetonate)diisopropoxide was obtained from Merck (Darmstadt, Germany), 4,4′-methylene diphenyl diisocyanate (MDI) was purchased from Fisher Scientific (Schwerte, Germany), and 1,4-butane diol (BD) was obtained from Alfa Aesar (Kandel, Germany). PPG-diol and BD were stored over a molecular sieve (Zeolite 4Å, obtained from Alfa Aesar, Kandel, Germany) for at least one day at 23 °C.

*Synthesis of thermoplastic polyurethane:* Following the prepolymer method, a PPG-based thermoplastic polyurethane was synthesized. To obtain a TPU with a hard segment content of 60 wt%, the stoichiometric ratio of the reactants PPG, MDI, and BD was set to 1.00:2.17:1.16. The reaction was carried out with a slight excess of isocyanate (NCO/OH = 1.005). The PPG-diol was heated to 125 °C and stirred under nitrogen flow. Afterward, two droplets of a catalyst solution consisting of PPG and 5 wt% of titanium(IV)bis(acetylacetonate)diisopropoxide were added. After a few seconds, MDI was added and the mixture was further heated to 155 °C and held there under continuous stirring for about 5 min. The obtained prepolymer was directly brought to reaction with BD, which serves as a chain extender. In parallel, the stirring speed was increased. As soon as the viscosity rose significantly, the reaction was stopped, and the melt was poured onto a plate covered with a foil of polytetrafluoroethylene. Finally, the obtained TPU was cured in an oven at 80 °C for 120 min.

*Post-processing of the TPU:* The synthesized TPU was ground with a cutting mill of type M 50/80 from Hellweg Maschinenbau (Roetgen, Germany). The obtained flakes served as raw material for further processing. 

*Extrusion:* The TPU flakes were dried at 110 °C overnight in a vacuum drying chamber, VDL 53, from Binder GmbH (Tuttlingen, Germany) and fed into a twin screw extruder, MICRO 18 GL, from Leistritz AG (Nürnberg, Germany) to produce filaments with a diameter of 2.85 mm ± 0.10 mm. The temperatures of the individual heating zones of the extruder were 200, 200, 202, 203, 190, 180, and 180 °C along the extrusion direction. The same extrusion line was used as earlier described [54]. 

*Additive manufacturing*: The filament was further processed via fused filament fabrication (FFF) to manufacture samples, such as tensile bars of type 5B [55], whose dimensions only deviated from the ideal dimensions by about ±0.1 mm due to the employed additive manufacturing method. For FFF, an Ultimaker 3 from Ultimaker B.V. (Utrecht, The Netherlands) and the slicing program Cura 4.8.0 [56] were used. The nozzle temperature was set to 200 °C, the build plate temperature to 65 °C, and the printing speed to 50 mm·s^−1^. 

*Injection molding:* The TPU flakes were injection molded into foil by Nea Kuasu Mold Tech GmbH. The barrel heating was set to a temperature between 210 °C and 225 °C. The injection pressure was approximately 470 bar, the injection time was 500 ms, the holding pressure was 300 bar, the holding pressure time was 4 s, and the cooling time was 30 s at a temperature of 40 °C. Finally, foil with a thickness of 1.00 mm ± 0.04 mm and a diameter of 12.50 mm ± 0.01 mm was obtained. The foil served as mold blanks for the thermoforming process to produce aligners, as described below.

*Fourier-transform infrared (FT-IR) spectroscopy:* The synthesized TPU was investigated by FT-IR spectroscopy. The measurement was carried out with a Nicolet Nexus 470/670/870 FT-IR spectrometer from Thermo Fisher Scientific (Madison, WI, USA) utilizing an attenuated total reflectance device. The FT-IR spectrum was measured from 4000 cm^−1^ to 650 cm^−1^ and averaged from a total of 40 scans with a spectral resolution of 2 cm^−1^.

*Characterization of thermal properties:* The phase transitions of the TPU were characterized by DSC using a Q100 DSC from TA Instruments (New Castle, DE, USA). The experiment was conducted on about 5 mg of the raw material that was immediately obtained after polymer synthesis. The sample was heated from 0 to 100 °C before it was cooled back to 0 °C. The whole thermal cycle was carried out twice. Both for cooling and heating, a rate of 10 °C·min^−1^ was applied. The temperature holding time at the minimum and maximum temperature was 2 min each. The glass transition temperature was determined for the second heating cycle as the inflection point using the standard analyzing software of the calorimeter. To study the thermal properties after water storage, tensile bars of type 5b [55] as obtained after FFF (see above) were put in a vessel filled with water. The vessel was heated to 37 °C and the temperature was maintained for three days with a heating plate. Then, the tensile bars were removed from the water and their surface was dried with a tissue before thermal and thermomechanical analyses were conducted. For a DSC measurement, a sample with a weight of about 5 mg was cut out of a tensile bar, while other tensile bars were thermomechanically analyzed by DMA. The characterization was conducted with the same methodologies and parameters as described above, except for the evaluation of the glass transition temperature in the DSC measurement, which was determined for the first heating cycle.

*Characterization of thermomechanical properties:* The thermomechanical properties of the synthesized and water stored TPU were studied on the center part of an additively manufactured type 5B tensile bar [55]. The experiments were carried out in multifrequency–strain mode with a Q800 DMA from TA Instruments (New Castle, DE, USA) using film tension clamps. A frequency of 10 Hz, a static force of 0.1 N, and an oscillating amplitude of 10 μm were selected. At first, the sample was cooled to 0 °C and held there for 5 min before it was heated to 100 °C with a rate of 3 °C·min^−1^ while the storage modulus, loss modulus, and loss factor were recorded.

*Characterization of shape memory properties:* The shape memory properties of the synthesized TPU were studied in a cyclic thermomechanical measurement using the Q800 DMA from TA Instruments (New Castle, DE, USA). Again, a tensile bar type 5B [55] was additively manufactured and its center part was fixed in the film tension clamps of the DMA device. Using the strain rate mode of the DMA, the following program was executed. First, the sample was heated to *T*_high_ = 80 °C followed by a holding time of 25 min. Then, the sample was stretched to a maximum strain *ε*_max_ of 100% with a rate of 50%·min^−1^. After 30 s, the sample was cooled to *T*_low_ = 23 °C, where it was held isothermally for another 20 min. Adjacently, the force was set to 0 N in order to release the stress inside the sample within 10 min to finalize the thermomechanical treatment (programming). The temporary shape was determined with regard to the fixed strain *ε*_u_. Lastly, the shape memory effect (SME) was triggered by heating the sample to 80 °C and subsequently holding the temperature for 25 min. After cooling to 23 °C and an isothermal holding time of 10 min, the permanent shape was recovered and the strain *ε*_r_ determined. The procedure was repeated for another four cycles. In all cases, the heating and cooling rate was 5 °C·min^−1^. To evaluate the shape memory properties, Equation (1) was used to determine the strain fixity ratio *R*_f_(N) as an indicator for how well the polymer can retain the imposed temporary shape in the Nth cycle. Equation (2) was used to determine the cycle-related strain recovery ratio *R*_r_(N) as an indicator of the polymer’s ability to recover the original shape.

(1) $$R_{f}\left(N\right)=\frac{\varepsilon_{u}}{\varepsilon_{max}}$$

(2) $$R_{r}\left(N\right)=\frac{\varepsilon_{max}-\varepsilon_{r}(N)}{\varepsilon_{max}-\varepsilon_{r}(N-1)}$$

*Cytotoxicity test:* The biocompatibility of the TPU was investigated by Bioserv Analytik-und Medizinprodukte GmbH. The tests were conducted according to DIN EN ISO 10993-5:2009-10 [57]. Mouse fibroblast cells in the form of ATCC CCL 1, NCTC Clone 29 were used for the cell toxicity test. The cultivation medium was Dulbecco’s modified Eagle Minimum Essential Medium (DMEM) with 10% fetal calf serum (FCS). Cell cultivation was performed at 37 °C in a carbon dioxide incubator (5% of CO_2_) for 24 h to produce a subconfluent monolayer. The cell cultures were checked for subconfluence and morphology. After the cultivation medium had been removed, a sample of TPU was incubated together with the monolayer at 37 °C in a carbon dioxide incubator (5% of CO_2_). After 24 h of exposure, the cell layer was microscopically investigated in order to identify cytoplasmic damage. The result was compared to the positive and negative control. Aseptic polypropylene was used as a negative sample and polyurethane, which was verified to be cytotoxic, was used as a positive sample. Both materials were treated in the same way as the TPU.

*Characterization of water absorption:* The water absorption of the TPU was measured gravimetrically. Samples weighing 615.1 mg ± 0.2 mg (*m*_dry_) in their dry states were stored for 3 days in 37 °C water, whereupon their weight increased to 629.2 mg ± 0.2 mg (*m*_wet_). For each sample, the average value of three measurements was determined. The relative water uptake was calculated from the difference in the mass of the wet sample (*m*_wet_) and the dry sample (*m*_dry_) according to Equation (3).

(3) $$Water\;uptake=\frac{m_{wet}-m_{dry}}{m_{dry}}$$

*Qualitative analysis of shape memory effect in water:* An approximately 5 cm long piece of extruded filament with a diameter of 2.85 mm was heated from 23 °C to 80 °C in a convection oven (UF110 from Memmert GmbH + Co. KG (Schwabach, Germany)) and remained at this temperature for about 5 min. The ends of the hot filament were manually pushed together whereupon the filament bent to achieve a circular shape before it was cooled to room temperature to fix the temporary shape. After 5 min, the programmed sample was placed in a water bath pre-tempered to 37 °C. The temperature was maintained for 48 h with a heating plate. Pictures were taken at regular intervals with a camera to follow the shape recovery behavior of the sample.

*Cyclic DMA measurement:* The feasibility of the SMP for staging was evaluated in a cyclic DMA measurement on a tensile bar of type 5b [55]. Again, the Q800 DMA from TA Instruments (New Castle, DE, USA) was used. T_high_ was set to 80 °C and *T*_low_ was set to 23 °C. The isothermal holding times for both *T*_high_ and *T*_low_ were 20 min with an extended temperature holding time of 10 min after stretching at *T*_high_, as well as 10 min after stress release at *T*_low_. After triggering the SME, the sample was cooled back to *T*_low_ and held there for 10 min to complete the cycle. After every two cycles, *ε*_m_ gradually increased to 25%, 50%, 100%, and 200%. In any case, a temperature rate of 5 °C · min^−1^ and a strain rate of 25%·min^−1^ were applied. The strain fixity ratio *R*_f_(N) and the strain recovery ratio *R*_r_(N) were also calculated for each cycle according to Equations (1) and (2), respectively.

*Computer-aided design and additive manufacturing of denture models:* In order to facilitate further processing of the SMP foil into aligners, a denture model was created virtually. It was meant to later function as a form-giving structure in the thermoforming process of the SMP foil. An ideal digital dental model was kindly provided by CruisAIDer3D GbR. Archform software 1.8 [58] allowed a virtual simulation of tooth malocclusion, which was initially carried out as an extreme proclination of an upper incisor. The ideal and the transformant dental models were then additively manufactured with a DLP printer using ‘Sprintray Die and Model’ resin as print material. Additionally, using the open-source software Blender [59], the malpositioned incisor was separated from the denture and both parts were additively manufactured again. The single tooth model and the remaining ideal dental arch model were necessary to subsequently demonstrate the singular movement of the tooth caused by the aligner.

*Thermoforming and programming of aligners:* Four aligners were produced with the pressure molding machine Biostar^®^ VII for applications in dental pressure molding and orthodontics, which was kindly provided by Scheu-Dental GmbH (Iserlohn, Germany). Therefore, the injection molded foil of the TPU was fixed in the Biostar^®^ device and heated with the standard proportional-integral-derivative parameters of the device for 35 s, since this time was judged to be suitable for thermoplastic forming of the material. The selected time frame was neither too long for deliquescence nor too short for programming to occur. The softened foil was thermoformed onto the model denture without malocclusion (targeted tooth position) and cooled for 90 s by the intrinsic air cooling system of the Biostar^®^ device. The thermoformed foil was detached and a multifunctional rotary tool Dremel model 3000 equipped with a blade from Dremel (Konijnenberg, The Netherlands) was used to cut out the aligner. In total, four aligners characterized by a rough dimensioning of 66.5 mm (maximum distance between the molar teeth) × 59.5 mm (length of a line that begins between the two incisors and ends where it centrally intersects the line connecting the two molars) × 15.0 mm (maximum aligner height), as determined with a caliper gauge from Fowler High Precision GmbH (Canton, MA, USA), were produced. In a thermal pretreatment, the four aligners were annealed for 5 min at 70 °C in a convection oven (UF110 from Memmert GmbH + Co. KG (Schwabach, Germany)). Then, every single aligner was manually placed on the denture model with a misaligned incisor (actual tooth position). The denture model, mounted with the aligner, was cooled to 23 °C. After 5 min, the aligner was removed in order to obtain it in its programmed shape.

*Characterization of thermo-responsiveness:* To investigate thermo-responsiveness, a misaligned incisor was cut out of the denture model using a Dremel model 3000 multifunctional rotary tool equipped with a blade from Dremel (Konijnenberg, The Netherlands). The tooth was loosely reinserted and the programmed aligner was attached to the denture. The aligner was heated to 80 °C in the temperature chamber of a Criterion universal testing machine (model 43) from MTS Systems Corporation (Eden Prairie, MN, USA). The material behavior when triggering the SME was monitored by recording a video.

In another approach, an artificial tooth with a 12.5 mm long pin was loosely inserted in the malocclusion of the second programmed aligner and was attached to the denture. Again, the demonstrator was heated to 80 °C in the climatic chamber and a video was recorded. By overlaying the pictures before and after triggering the shape memory effect, the position of the pin with regard to its angle movement was graphically evaluated using CorelDraw 2019 [60]. The absolute displacement was then calculated to be 3.5 mm as the arc length *S* using the following Equation (4):

(4) $$S=\frac{\pi \times r\times \theta }{180{}^{\circ}}$$

with *r* being the total length of the tooth (9.12 ± 0.01 mm) and *θ* the measured angle of the movement (22°).

In a third case, the shape recovery force of an aligner was investigated. Again, the programmed aligner with a misaligned incisor was manually put on the denture model exhibiting the loose incisor. The aligner was clamped to a standing tool angle. A Vicat needle with an attached steel ball with a diameter of 7 mm was mounted to the force sensor of the Criterion universal testing machine (model 43) from MTS Systems Corporation (Eden Prairie, MN, USA). The tip was brought so close to the aligner that it barely touched the misaligned incisor. The aligner was heated with an infrared emitter type IRD S250G from Optron (Garbsen, Germany) in order to trigger the shape memory effect. During shape recovery, the released force was recorded by the sensor.

*Characterization of water-responsiveness:* To investigate water-responsiveness, the same set-up containing a loosely inserted tooth in a denture model, which was used to study thermo-responsiveness, was selected. In this case, the fourth programmed aligner was put into 37 °C water. The temperature of the water was maintained for 20 h with a heating plate and pictures were taken in regular time intervals with a photo camera to follow shape recovery.

## 3. Results and Discussion

**Figure 1:**
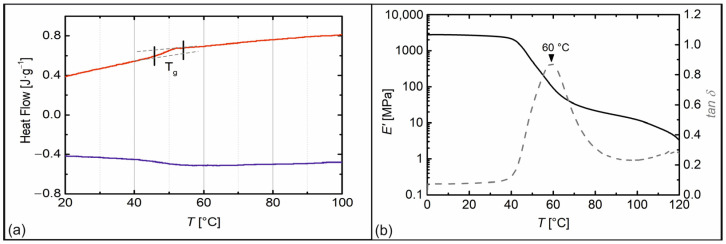
Thermal and thermomechanical properties of PPG-based TPU as determined by DSC (**a**); second heating (red) and cooling (purple) at heating and cooling rates of 10 °C·min^−1^) and DMA (**b**); temperature dependence of storage modulus *E′* (solid line) and tan δ (dashed line) at a heating rate of 3 °C·min^−1^).

For their therapeutic use, aligners must have suitable thermomechanical properties. In addition, it is advantageous for aesthetic reasons if they are clear and virtually almost invisible. For these reasons, a thermoplastic polyurethane (TPU) having a non-crystalline, amorphous soft segment of polypropylene glycol (PPG) with a low molecular weight of about 430 g·mol^−1^ was selected. The counterpart was the hard segment, which was obtained from the reaction of 4,4′-diphenylmethane diisocyanate (MDI) and 1,4-butanetiol (BD). The ratio of hard to soft segments was 0.6/0.4. The prepolymer method was used to synthesize a similar poly(ether urethane) as recently developed for four-dimensional (4D) printing to enable thermally inducible assembly processes [54]. Fourier-transform infrared (FT-IR) spectroscopy was employed to verify the completeness of the polyaddition reaction (Figure S1, Supplementary Materials). Here, the characteristic vibrational modes of a poly(ether urethane) were detected. In addition, only a weak signal at 2270 cm^−1^ associated with the presence of freely available isocyanate appeared in the spectrum, indicating that the reaction was largely completed. Differential scanning calorimetry (DSC) and dynamic mechanical analysis (DMA) were used to study the thermal behavior, as well as the temperature-dependent mechanical properties of the TPU (Figure 1).

**Figure 2:**
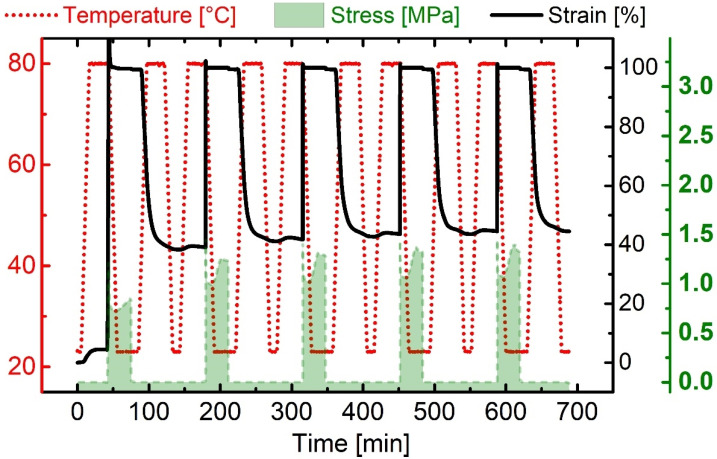
Cyclic DMA measurement protocol for PPG-based TPU. The individual parameters included *T*_high_ = 80 °C, *T*_low_ = 23 °C, and *ε*_max_ = 100%. The evolution of temperature (red dotted), stress (green area), and strain (black line) were recorded for five cycles.

In the DSC thermogram (Figure 1a), a phase transition between 45 °C and 55 °C with an inflection point at about 50 °C could be detected and assigned to the glass transition temperature *T*_g_ of the TPU. The result was confirmed by the DMA measurement (Figure 1b). Here, a maximum for tan δ occurred at about 60 °C (Figure 1b, dashed line). As known from other urethane-based SMPs, *T*_g_ is a few degrees higher in DMA measurements compared to the DSC results, which is due to the different testing procedures and polymer chain dynamics under the respective testing conditions [23,61]. When passing through the glass transition, a one-step decrease in storage modulus *E’* occurred, associated with a softening of the TPU (Figure 1b, solid line). Afterward, the material entered a soft rubbery region, indicated by the almost linear evolution of *E*′ with a slightly negative slope. As essential for the envisaged application as an orthodontic aligner, the TPU was transparent, had good mechanical stability at 23 °C (see *E*′ in Figure 1b), and had a glass transition just above human body temperature. The results were similar to a previous study, in which the material had been used for four-dimensional printing [54]. Minor differences in physical properties can be explained with batchwise synthesis. To investigate the strain-related shape memory properties of the TPU, a cyclic DMA measurement was conducted (Figure 2).

**Figure 3:**
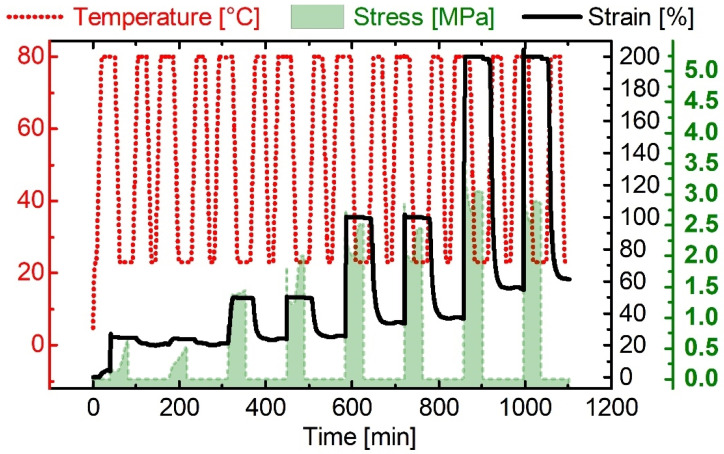
Cyclic DMA measurement protocol for PPG-based TPU (*T*_high_ = 80 °C, *T*_low_ = 23 °C, *ε*_max_ = 25%, 50%, 100%, and 200%). The evolution of temperature (red dotted line), stress (green area), and strain (black line) are exhibited.

The material was first heated above *T*_g_, stretched, and cooled below *T*_g_ while maintaining the applied force. After unloading at 23 °C, the polymer was in its temporary, so-called programmed shape. At this stage, it was thermo-responsive—heating above *T*_g_ triggered the SME and the TPU recovered much of the previously applied strain. Based on the experiment, the strain fixity ratio *R*_f_(N) and the strain recovery ratio *R*_r_(N) were determined. In every single thermomechanical cycle, the synthesized PPG-based TPU exhibited an excellent strain fixability *R*_f_(N = 1–5) > 99%. In the first deformation, distinct reorientation processes are known to occur in physically cross-linked urethane-based SMPs [62,63,64]. Therefore, only a low value of 63% could be determined for *R*_r_(N = 1). However, from the second cycle onwards, the TPU exhibited an excellent strain recovery ratio *R*_r_(N = 2–5) exceeding 94%. The cycle-related measurement data can be extracted together with their evaluation from Supplementary Materials (Table S1). The shape memory behavior of the TPU was examined in another cyclic DMA measurement for various deformations. Again, the deformation temperature and the upper temperature to trigger the shape memory effect were set to 80 °C, while the lower temperature remained the same. The thermomechanical treatments and the conditions to trigger the shape memory effect were adjusted by raising the maximum strain applied after every second cycle. The measurement protocol is exhibited in Figure 3.

Independent of the applied strain, the SMP showed very good strain fixity ratios (*R_f_*(N) > 96%). In the first cycle at *ε*_max_ = 25%, the strain recovery ratio *R_r_*(N) was again low due to reorientation processes in the TPU [65]. Upon increasing the maximum strain applied, the SMP achieved values for *R*_r_(N) exceeding 88%. The cycle-related data can be extracted from the Supplementary Materials (Table S2). In other words, this implies that when an aligner made from the introduced TPU is reprogrammed with drastic or even less severe deformations, it may still exhibit promising shape memory properties. This opens up the possibility to reuse the aligner for changing deformation scenarios. Furthermore, the stresses (green area), which are stored inside the material during programming and are released while triggering the shape memory effect, vary with different deformations. Hence, the exerted forces of an aligner made from the TPU can be precisely adjusted. Thus, a first intermediate goal could be achieved for the TPU against the background of its applicability for orthodontic aligners.

**Figure 4:**
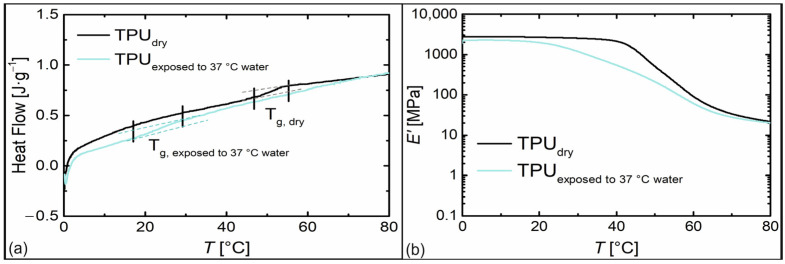
Thermal and thermomechanical properties of PPG-based TPU immediately after synthesis (black) compared to the same material stored in water for 3 days at 37 °C (light blue). DSC (**a**); second heating with rates of 10 °C·min^−1^) and DMA (**b**); temperature dependence of storage modulus *E*’ at a heating rate of 3 °C·min^−1^).

In the next step, the influence of storage conditions in 37 °C water on the material was investigated. In this regard, water uptake of the TPU by 2.3 wt% was verified after three days. Subsequently, DSC and DMA measurements were conducted to analyze the impact on thermal and thermomechanical properties (Figure 4).

**Figure 5:**
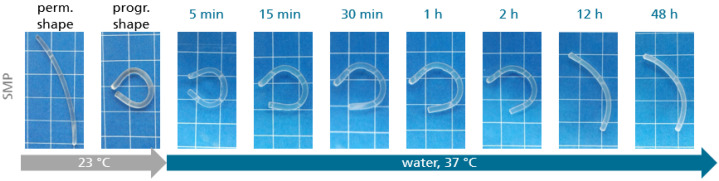
Water-responsiveness of a piece of filament from PPG-based TPU. From left to right: permanent shape at 23 °C, temporary shape at 23 °C, time-lapse activation of the shape memory effect in 37 °C water. For visualization, centimeter paper is shown in the background of the individual images.

Due to the exposure to water, the glass transition temperature of the TPU broadened and was shifted to lower temperatures to a region between 17 °C and 29 °C (Figure 4a). At the same time, the storage modulus *E′* decreased from 2300 MPa to 690 MPa at 37 °C (Figure 4b). As is known from many polymers, water acts as a plasticizer [66], which can even be used to trigger the SME in polyether-based TPUs, when the glass transition temperature drops below the storage temperature [67,68]. To investigate the water-responsiveness of the SMP, a piece of its filament was thermomechanically treated, and the programmed sample was stored in 37 °C water (Figure 5). Due to the shift in glass transition, the shape memory effect could be triggered slowly within a time period of about 12 h.

In fact, large parts of the permanent shape could be recovered within 12 h of water exposure. The residues still present in the material at this time were not restored even after 48 h. The slow shape recovery implies that the stresses stored in the material could be moderately released. This might be advantageous for an application as aligner material if it is possible to prevent an immediate recovery and thus the occurrence of riskily high initial forces.

To estimate the suitability of the TPU for its in vivo application, an in vitro cytotoxicity test was performed [57]. Therefore, mouse fibroblast cells were incubated together with the TPU and subconfluent monolayers of the cells were examined microscopically for cytoplasmic damage afterward. Against this background, the synthesized TPU did not cause any biological toxicological damage to the cells and can be classified as non-cytotoxic [69].

**Figure 6:**
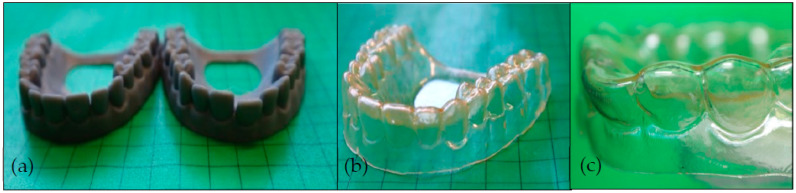
Digitally modeled and 3D-printed denture without malocclusion (**a**, left) and with misaligned incisor (**a**, right) and the corresponding aligner with targeted tooth position deep-drawn with SMP via thermoforming (**b**) and close-up of the programmed aligner with incisor malocclusion (**c**).

To demonstrate the principle of thermally switchable aligners, the SMP was injection molded and the resulting foil was used for thermoforming. As a counterpart, two dentures were fabricated via digital light processing (Figure 6). In detail, a denture model without malocclusion (targeted tooth position, Figure 6a, left) and a denture model with a misaligned incisor (actual tooth position, Figure 6a, right) were additively manufactured. Subsequently, the injection molded foil was deep-drawn (thermoformed) over the model shown in Figure 6a, left, to obtain an aligner exhibiting the optimum tooth position with the SMP being in its permanent shape (Figure 6b). Due to the amorphous soft segment of the TPU, the aligner was clear and not opaque. Subsequently, the aligner was heated above the glass transition temperature of the TPU and manually placed on the denture with a misaligned incisor (Figure 6a, right). After cooling to 23 °C, the temporary shape was fixed and a programmed aligner exhibiting the incisor malocclusion was received (Figure 6c).

**Figure 7:**
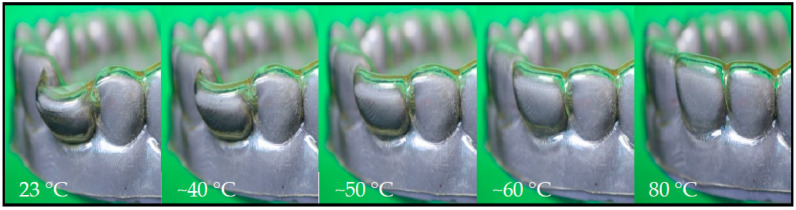
Heating-induced movement of a loose incisor from misaligned to its ideal position as enabled with a TPU aligner by the thermally triggered SME. The temperature gradually increased from 23 °C (left) to 80 °C (right).

Now, the aligner was placed on a model denture with a loose incisor at the same position, at which the model showed tooth misalignment (Figure 6a, right), and was heated to 80 °C. The thermo-responsiveness of the aligner is shown in the image series of Figure 7 and the corresponding Video S1 movie in the Supplementary Materials.

When heated to 80 °C, the SME was triggered and thus the dental misalignment was corrected by changing the position of the loose incisor. It is noteworthy that interrupting the heating by temporarily keeping the temperature constant led to the stabilization of the shape present at that time, which showed that it is also possible to gradually recover the shape of the aligner if a stepwise change of the aligner’s shape is envisioned. This means that heating to 80 °C is not necessarily required with the material used, so a temperature between 50 °C and 60 °C, which may be perceived as pleasant for a patient, could already be sufficient to induce significant shape recoveries. Moreover, the thermo-responsive material behavior agreed with the position of the signal for tan δ in the DMA associated with the glass transition temperature range (Figure 1b).

**Figure 8:**
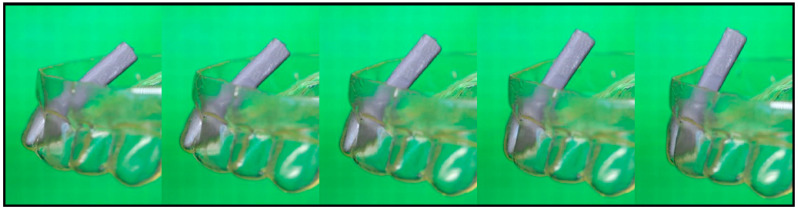
Thermo-responsiveness of a programmed TPU aligner with a misaligned incisor. The pin was inserted to better follow shape recovery on heating from 23 °C (left) to 80 °C (right).

To study the extent of the tooth movement in more detail, an artificial tooth with a pin was fabricated and inserted into another programmed aligner (Figure 8).

**Figure 9:**
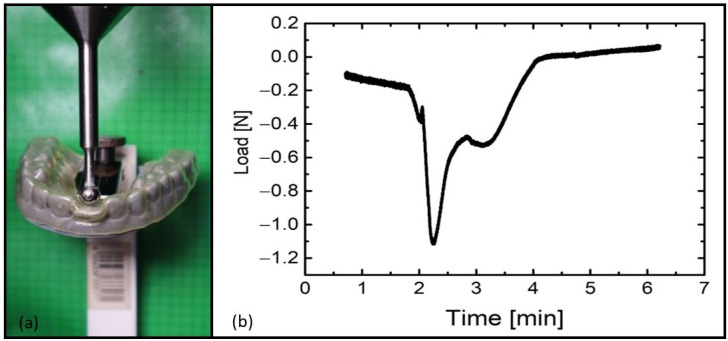
Experimental setup to determine the shape recovery force of a TPU aligner (**a**) and the corresponding measurement curve (**b**).

As a result of heating from 23 °C to 80 °C, the aligner corrected the displacement of the incisor while the position of the pin changed (Video S2). By subtracting the angles of the pin position in the temporary shape of the aligner (Figure 8, left) and in its recovered shape (Figure 8, right), a tipping angle of 22° could be estimated. Therewith, the incisor movement and correction of the displacement could be calculated by an arc length of about 3.5 mm. Although the incisor to be moved in the denture model did not offer significant resistance when the shape memory effect was thermally triggered, it can be assumed that the technology may also enable tooth movement in vivo [51]. To obtain a first indication of this, another experiment was conducted (Figure 9). The shape recovery force was measured with the force sensor of a tensile testing machine, which touched the programmed aligner (Figure 7, left) at the position of the misaligned tooth, and changes in axial load were followed (Figure 9a). It turned out that the aligner exerted a force of approximately 1.1 N when triggering the SME (Figure 9b).

Since shape recovery is a geometrically complex and not a uniform three-dimensional process, the measurement showed an immediate drop of the force after reaching 1.1 N, as well as a dip after 3 min at approximately 60 °C (Figure 9b). Thus, the measured recovery force gave a first approximation of the functionality of the aligner. It is noticeable that the maximum force was relatively large. However, the usual force for torquing a central incisor is approximately 1 N [70], which matched with the measured force of the TPU aligner. Basically, if desired, there are two ways of reducing the applied force. The reduction in foil thickness could be as helpful as gradual heating, at which the latter may result in a gradual increase in shape recovery force. Moreover, the water-responsiveness of the TPU could offer key advantages, particularly because water absorption is usually accompanied by a decrease in polymer stiffness.

**Figure 10:**
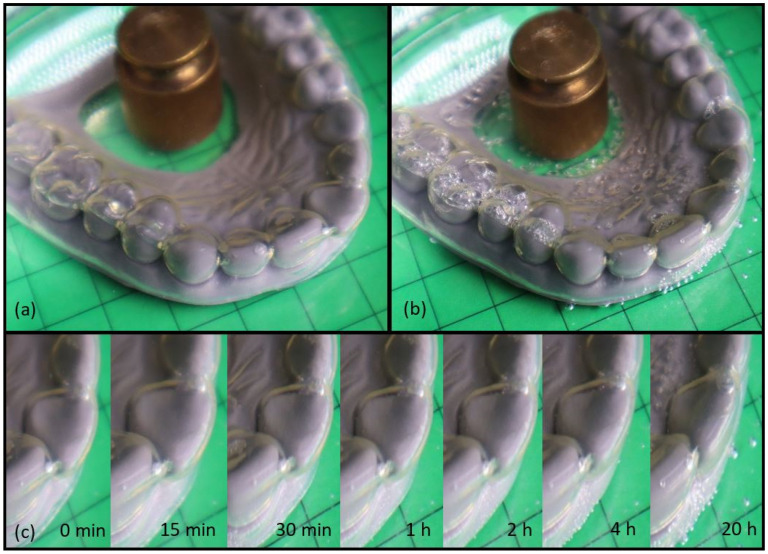
Water-responsiveness of a programmed TPU aligner with a misaligned incisor in 37 °C water (the weight prevented buoyancy). (**a**) A programmed aligner (0 min in a water bath), (**b**) a recovered aligner after storage for 20 h in a water bath, (**c**) a time-lapse activation of the shape memory effect depicted by the enlarged section of the incisor.

To gain deeper insights into material behavior, the water-responsiveness of a programmed aligner was studied (Figure 10). The programming procedure was the same as for the thermo-responsive aligner, but this time the programmed aligner was placed into a 37 °C water bath together with the model denture exhibiting the loose incisor.

It can be clearly seen that recovery into the permanent shape was possible within 20 h. It is noteworthy that the release of programmed recovery forces directly in the oral cavity by contact with water, but possibly also by saliva, appears to be an interesting alternative to conventional approaches in aligner orthodontics and requires further detailed investigation, e.g., the interaction of saliva with the material used. The detected water-responsiveness roughly correlated to the qualitative experiment on the filament (Figure 5). The fact that the introduced SMP is both thermo- and water-responsive is a significant advantage compared with the material behavior of standard polymers and leaves plenty of room for conceptual expansion in the field of aligner orthodontics.

## 4. Conclusions

This work aimed to synthesize and process a thermoplastic polyurethane, which is not cytotoxic and both thermo- and water-responsive, to gain initial insights into its applicability in orthodontic aligner therapy. Apart from the proven non-cytotoxicity and dual stimuli-responsiveness, gradual switching of an aligner could be achieved. Although the current approach requires one dental arch to be printed for the current malocclusion and another one for the desired tooth position, the introduced dual stimuli-responsive aligner technology is particularly promising from a sustainability point of view. The step-by-step movements may lead to a reduction in the number of setup steps and the number of aligners needed in therapy. This could save material costs and time in the future. The next essential steps are to measure water-responsiveness with regard to force recovery, to investigate combinatorial approaches by triggering partial shape recovery via heating to fine-tune a temporary shape, followed by triggering shape recovery in water, and to study the durability of the thermoplastic polyurethane, as well as to perform a detailed biomechanical analysis. In the case of the latter, an artificial tooth can be used as a link to a moment-force sensor to investigate the extent of forces and moments acting on teeth while being moved when heating a programmed aligner. The moment-force sensor will, therefore, be attached to a six-axis robot arm to react to the applied external stimulus and evaluate the consistency of the SMP characteristics by software specifically programmed for this purpose. In this way, it will be possible to determine the degree of staging that can be incorporated into future aligners without wearing away their features or damaging the sensitive periodontal tissue. All in all, the promising functionality of the introduced material can lead to novel approaches in aligner therapy.

## 5. Patents

Due to the particular economic attractiveness of the technology presented here, parts of this work were protected in advance of this publication within the framework of a patent filing.

## Data Availability

The data presented in this study are available in the Supplementary Materials. The authors will be happy to provide information on the biological evaluation of the TPU investigated here with regard to its in vitro cytotoxicity upon request.

## Supplementary Materials

The following supporting information can be downloaded at: https://www.mdpi.com/article/10.3390/ma16083094/s1. References [71,72,73,74,75] are cited in the supplementary materials.
