# Supplementary material for: Dual Stimuli-Responsive Orthodontic Aligners: An In Vitro Study
Source: Materials (Basel). 2023 Apr 14;16(8):3094. doi: 10.3390/ma16083094 (PMC10145520; doi:10.3390/ma16083094)
Supplement: Supplementary file 1 [file materials-16-03094-s001.zip › materials-2218668-supplementary.pdf]

Article

Dual Stimuli-Responsive Orthodontic Aligners: An In Vitro Study

Fourier-transform infrared (FTIR) spectroscopy

In the FT-IR spectrum of the synthesized TPU, a very weak signal occurred at about 2270 cm^{-1}, indicating that only a few free isocyanate groups were available [1]. This speaks to an almost complete reaction. At the same time, vibration modes are present that are characteristic of the formation of a poly(ether urethane) (Figure S1).

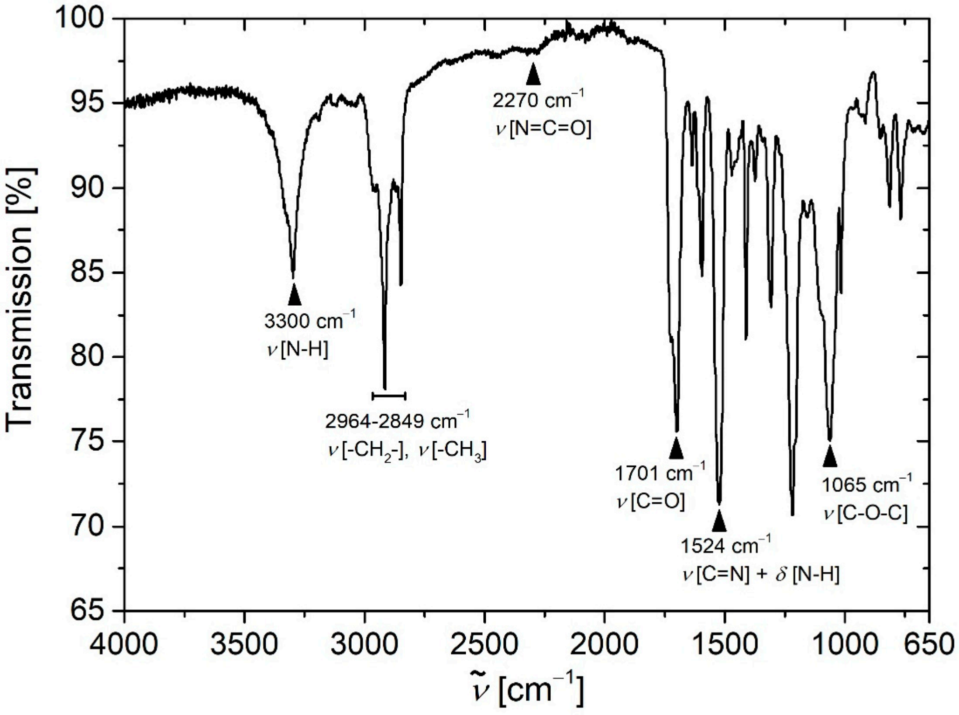

Figure S1. FT-IR spectrum of polypropylene glycol (PPG)-based poly(ether urethane), including the assignment of vibration modes and the specification of their wavenumbers.

A more detailed analysis of the FT-IR spectrum shows overlapping absorbances between 2964 cm^{-1} and 2849 cm^{-1}, which can be assigned to asymmetric and symmetric stretching vibrations of CH_2 alkyl entities together with CH_3 entities of the PPG side chain [2,3]. The absorbance band at 1065 cm^{-1} can be assigned to the ν[C–O-C] stretching vibrations of the PPG soft segment [3,4]. The stretching vibrations ν[N–H] at 3300 cm^{-1}, ν[C=O] in the carbonyl stretching region at 1701 cm^{-1}, as well as an amide peak (ν[C=N] + δ[N–H]) at 1524 cm^{-1}, indicate the formation of the hard segment. The signals can be attributed to the formation of urethane species [3,5,6]. As a result, the reaction seemed to be successful.

Cyclic thermomechanical measurements

Table S1. Dependence of shape memory properties of the synthesized TPU on cycle number. For T_{low} and T_{high}, 23 °C and 80 °C were selected, respectively, while ε_{max} was set to 100%.

Cycle number N	Fixed strain ε_u	Recovered strain ε_r	Shape fixity ratio $R_f(N)$	Shape recovery ratio $R_r(N)$
**1**	100%	37%	>99%	63%
**2**	100%	41%	>99%	94%
**3**	100%	43%	>99%	97%
**4**	100%	44%	>99%	98%
**5**	100%	45%	>99%	98%

Table S2. Dependence of shape memory properties of the synthesized TPU on ε_{max}. For T_{low} and T_{high}, 23 °C and 80 °C were selected, respectively.

Maximum strain applied ε_{max}	Cycle number N	Fixed strain ε_u	Recovered strain ε_r	Shape fixity ratio $R_f(N)$	Shape recovery ratio $R_r(N)$
**25%**	1	25%	20%	>99%	20%
	2	24%	20%	96%	>99%
**50%**	1	50%	23%	>99%	90%
	2	50%	25%	>99%	93%
**100%**	1	100%	34%	>99%	88%
	2	100%	37%	>99%	95%
**200%**	1	199%	56%	>99%	88%
	2	199%	61%	>99%	97%

References

1. Sáenz-Pérez, M.; Lizundia, E.; Laza, J.M.; García-Barrasa, J.; Vilas, J.L.; León, L.M. Methylene diphenyl diisocyanate (MDI) and toluene diisocyanate (TDI) based polyurethanes: thermal, shape-memory and mechanical behavior. *RSC Adv.* **2016**, *6*, 69094–69102, doi:10.1039/c6ra13492k.
2. Erdem, A. Synthesis and characterization of polypropylene glycol-based novel organogels as effective materials for the recovery of organic solvents. *J Appl Polym Sci* **2021**, *138*, 49997, doi:10.1002/app.49997.
3. Tan, C.; Tirri, T.; Wilen, C.-E. Investigation on the Influence of Chain Extenders on the Performance of One-Component Moisture-Curable Polyurethane Adhesives. *Polymers (Basel)* **2017**, *9*, doi:10.3390/polym9050184.
4. Zhang, Y.; Qi, Y.-h.; Zhang, Z.-p. The influence of 2,4-toluene diisocyanate content on the intrinsic self-healing performance of polyurethane at room-temperature. *J Polym Res* **2015**, *22*, doi:10.1007/s10965-015-0744-0.
5. Piril Ertem, S.; Yilgor, E.; Kosak, C.; Wilkes, G.L.; Zhang, M.; Yilgor, I. Effect of soft segment molecular weight on tensile properties of poly(propylene oxide) based polyurethaneureas. *Polymer* **2012**, *53*, 4614–4622, doi:10.1016/j.polymer.2012.08.020.
6. Pretsch, T.; Jakob, I.; Müller, W. Hydrolytic degradation and functional stability of a segmented shape memory poly(ester urethane). *Polymer Degradation and Stability* **2009**, *94*, 61–73, doi:10.1016/j.polymdegradstab.2008.10.012.
